# Elevated Levels of Cell-Free Circulating DNA in Patients with Acute Dengue Virus Infection

**DOI:** 10.1371/journal.pone.0025969

**Published:** 2011-10-07

**Authors:** Tran Thi Ngoc Ha, Nguyen Tien Huy, Lyre Anni Murao, Nguyen Thi Phuong Lan, Tran Thi Thuy, Ha Manh Tuan, Cao Thi Phi Nga, Vo Van Tuong, Tran Van Dat, Mihoko Kikuchi, Michio Yasunami, Kouichi Morita, Vu Thi Que Huong, Kenji Hirayama

**Affiliations:** 1 Department of Immunogenetics, Institute of Tropical Medicine (NEKKEN), Nagasaki University, Nagasaki, Japan; 2 Department of Virology, Institute of Tropical Medicine (NEKKEN), Nagasaki University, Nagasaki, Japan; 3 Laboratory of Arbovirus, Pasteur Institute, Ho Chi Minh City, Vietnam; 4 Children's Hospital No. 2, Ho Chi Minh City, Vietnam; 5 Center for Preventive Medicine, Vinh Long, Vietnam; 6 Global COE program, Nagasaki University, Nagasaki, Japan; Blood Systems Research Institute, United States of America

## Abstract

**Background:**

Apoptosis is thought to play a role in the pathogenesis of severe dengue and the release of cell-free DNA into the circulatory system in several medical conditions. Therefore, we investigated circulating DNA as a potential biomarker for severe dengue.

**Methods and Findings:**

A direct fluorometric degradation assay using PicoGreen was performed to quantify cell-free DNA from patient plasma. Circulating DNA levels were significantly higher in patients with dengue virus infection than with other febrile illnesses and healthy controls. Remarkably, the increase of DNA levels correlated with the severity of dengue. Additionally, multivariate logistic regression analysis showed that circulating DNA levels independently correlated with dengue shock syndrome.

**Conclusions:**

Circulating DNA levels were increased in dengue patients and correlated with dengue severity. Additional studies are required to show the benefits of this biomarker in early dengue diagnosis and for the prognosis of shock complication.

## Introduction

Dengue virus (DV), an arthropod-borne human viral pathogen with four distinct serotypes (DV-1, DV-2, DV-3, and DV-4), belongs to the genus *Flavivirus* of the family *Flaviviridae*. In the last two to three decades, dengue has become a major public-health burden in tropical and subtropical areas of the world, mostly in Southeast Asia and Western Pacific Regions. Dengue disease ranges from asymptomatic or self-limiting dengue fever to severe dengue characterized by plasma leakage (dengue hemorrhagic fever, DHF) that can lead to a life-threatening syndrome (dengue shock syndrome, DSS). Besides, severe dengue was also defined by severe bleeding, and/or severe organ impairment [1]. About 50–100 million dengue infections are estimated to occur annually, including about 250,000–500,000 cases of DHF/DSS [2]. So far, there is no effective vaccine or antiviral drug against the disease. Although mortality can reach 10–20% of severe cases [2], early appropriate treatment can reduce it to less than 1% [1]. Hence, the World Health Organization (WHO) encourages research on the development of new methodologies to serve as dengue severity markers [3]. In relation to this, a series of biological markers such as DV-nonstructural protein 1 (NS1), immune cytokines, and markers of endothelial cell damage and dysfunction have been investigated [4]. Remarkably, neopterin, which is synthesized by macrophages as an indicator of pro-inflammatory immune status, was reported to be a potential marker for severe dengue infection [5]. Furthermore, increased titers of soluble thrombomodulin [6], hyaluronan [7], or decreased levels of inter-α inhibitor proteins in dengue infection are also correlated with disease severity [8]. However, no effective marker for severe dengue prediction has been found so far [4].

The pathogenesis of DHF/DSS is still not completely understood. Various mechanisms have been suggested, including antibody-dependent enhancement in secondary dengue infection [9], memory T cell-mediated pathogenesis [10], suppressed Th1/predominant Th2 responses [11], cytokine tsunami [12], and anti-NS1 antibodies that cross-react with vascular endothelium [13]. Additionally, the virulence of the virus strain [9], [14] and host genetics [15] may influence the outcome of a dengue infection.

There is evidence of elevated circulating DNA levels in the plasma of patients with ovarian cancer [16], rheumatoid arthritis [17], and sepsis [18]. Apoptotic cells are suggested to be the main source for release of this cell-free DNA [19], which can activate innate immunity [20], [21]. In dengue infection, apoptosis could play a role in the pathogenesis of dengue severity. Apoptotic cell death following dengue infection is proposed to cause liver failure [22], damage to the central nervous system in a mouse model [23], and induce vascular permeability [24]. Furthermore, dengue infection has been shown to cause an increase in peripheral blood mononuclear apoptosis, which is also correlated with dengue severity [25]. However, to our knowledge, the increase of circulating human DNA in viral infectious diseases has not yet been investigated. Hence, we investigated the level of cell-free circulating DNA in acute DV infections, and its association with disease severity.

## Materials and Methods

### Study design

A hospital-based case control study was performed on children aged 6 months to 15 years with suspected dengue infections from 2006 to 2007 in the Children's Hospital No. 2 in Ho Chi Minh City and the Center for Preventive Medicine in Vinh Long province, Vietnam. This study was approved by the institutional ethical review committees of the Institute of Tropical Medicine, Nagasaki University, and the Pasteur Institute in Ho Chi Minh City. Informed consent was required for all subjects from the parents or guardians upon enrolment.

### Selection criteria

All of the dengue cases satisfied the 1997 WHO diagnostic criteria [26]: DF, an acute febrile with at least two of the symptoms such as headache, retro-orbital pain, myalgia, arthralgia, rash, hemorrhagic manifestations, or leukopenia; DHF with fever or history of acute fever lasting 2–7 days, bleeding signs, thrombocytopenia (100,000 cells per mm^3^ or less), or evidence of plasma leakage (hemoconcentration, pleural effusion, and ascites); DSS, symptoms of DHF plus shock signs including weak and rapid pulse, narrowing of the pulse pressure (<20 mm Hg), or hypotension with cold, clammy skin and restlessness. The extent of hemoconcentration was evaluated by comparing the maximum hematocrit to the minimum value recorded during hospitalization. A positive confirmed laboratory test was made when the result of dengue virus isolation was positive or RT-PCR assay determined a dengue serotype, or when there was a positive anti-DV IgM antibody-capture ELISA, a positive seroconversion, or a ≥4-fold increase in anti-DV IgG titres between acute and convalescent samples. The cases were diagnosed as secondary infection if the DV IgM/IgG ratio was <1.8 [27].

All patients hospitalized in the first 96 h of illness and corresponding to the above criteria were enrolled in the study. Patients who were suspected as dengue fever but had negative serological and virological diagnostic tests with dengue infection were defined as having other non-dengue febrile illnesses (OFIs). School children living in Ho Chi Minh City who had no symptoms of DV infection or other diseases, and tested negative in anti-DV IgM and IgG antibody-capture ELISA, were chosen as a healthy control group.

### Sample collection

Blood samples were drawn into EDTA tubes at the time of enrolment and in the convalescent phase prior to discharge from the hospital. Plasma was separated by centrifugation at 3000 rpm for 10 min and divided into two tubes: one used for dengue confirmation while the other was stored at −80°C and centrifuged again at 3000 rpm for 10 min before being used for cell-free DNA quantification.

### Dengue diagnosis

Dengue virus isolation using the C6/36 cell line as described previously [28], [29] and viral identification by a direct and indirect fluorescent antibody technique with monoclonal antibodies supplied by the Centers for Disease Control and Prevention (For Collins, CO, USA) [30], and molecular detection of the dengue virus genome by a Ready-To-Go reverse transcriptase PCR test kit (Amersham, MA, USA) [31], were carried out immediately on the acute plasma sample. Serological assays for anti-DV IgM and IgG by IgM-and IgG-capture ELISA (in-house Kit of the Pasteur Institute, HCMC) were carried out on both the acute and convalescent plasma samples, collected at ≥3-day intervals [27].

### Measurement of circulating DNA by Picogreen fluorometric degradation assay

The Quant-iT PicoGreen dsDNA Reagent and Kit was purchased from Invitrogen (Life Technologies Japan). Lambda DNA of known concentration was serially diluted and added to pooled plasma of 10 healthy donors with negligible DNA concentration to make standard DNA samples. Three microliters of standard DNA samples were diluted in 100 µl of TE buffer (10 mM Tris-HCl, 1 mM EDTA, pH 7.5) and incubated at 37°C for 1 h in the presence or absence of 6 µg bovine pancreatic DNase I (Sigma) activated in 10 mM MgCl_2_. In the preliminary test, more than 90% of added DNA was degraded in this experimental condition (data not shown). Then, an equal volume of the PicoGreen dye diluted 1∶200 in TE buffer was added to the reaction to make a final volume of 200 µl per well in a black 96-well microplate (Corning Japan, Tokyo, Japan). After incubation in the dark for 15 min, the fluorescent signal of the sample was measured at 485 nm excitation and 535 nm emission using Perkin Elmer ARVO™ MX-1420. Each sample was performed in duplicate. The reduction in fluorescent intensity between non-treated and DNase-treated samples was calculated, and then plotted against the standard DNA concentration to make a standard curve. Linearity was found within a DNA concentration range of 0–26667 ng/ml (Figure S1). The intra-assay coefficient of variation (CV) was 2.1–15.5%, while the inter-assay coefficient of variation was 0.8–23.8% (Table S1). The limits of quantification (LOQ) and detection (LOD) were 179 and 68 ng/ml, respectively. LOQ was determined as the mean concentration plus 10 standard deviations (SD) of a blank sample without DNA, and LOD was calculated by adding three SDs to the mean concentration of the blank. Hence, this linear line was used as a standard curve to estimate DNA amount (L') in 3 µl of plasma, and then the DNA level was expressed in nanograms per milliliter based on the following equation:
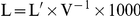
where L is concentration of DNA in plasma (ng/ml), and V is volume of plasma added to the reaction (3 µL).

The degradation fluorometric PicoGreen method was further validated by quantitative real-time PCR, which is considered to be the standard method for cell-free DNA quantification [16], [32]. A total of 84 random plasma samples from 20 healthy children and 64 dengue patients (22 DF, 30 DHF, 12 DSS) were selected for DNA extraction, and the amplification reaction followed a protocol previously published in which the copy number of the GAPDH gene was used to represent the DNA level in the plasma [16], [32] (at least 400 µl of plasma was used; details of the method are described in the Methods S1). The correlation between the two methods was analyzed by a Spearman rank test. Our results demonstrated that the fluorometric degradation method was highly correlated with real-time PCR (r = 0.78, P<0.0001) (Figure S2). This correlation coefficiency was higher than that of nucleosome-specific ELISA and real-time PCR, as previously reported [33]. Moreover, the PicoGreen method consumed very small volumes of plasma for each reaction, and had advantages of lower time, cost, and labor compared to the real-time PCR method. Hence, the degradation fluorometric PicoGreen assay was applied for all samples in this study.

### Statistical analysis

All statistical analyses were performed using STATA software, version 8.0. The Skewness and Kurtosis test was used for testing the normal distribution of continuous variables. Student's t test was used for continuous variables normally distributed, while Mann-Whitney tests were used for continuous variables which were not normally distributed. Correlation between the two methods was analyzed by a Spearman rank test. χ^2^ analysis was used for categorical variables. Fisher's exact test was used when the number was less than 5. The difference was considered significant at P<0.05. A multivariate logistic regression model was used to find the independent predictive value of the biomarker on disease outcome.

## Results

### Study population characteristics

A total of 281 subjects, including 194 dengue patients, 44 OFIs, and 43 healthy children were enrolled in this study, and their characteristics are summarized in Table 1. Among the 194 patients, 61 were DF, 76 were DHF, and 57 were DSS according to the 1997 WHO classification. There were no differences in sex and age between the control and dengue groups. The mean ages were lower in the DSS group compared to the DHF group (P<0.01) and lower in the OFI group compared to the DF group (P<0.01). For the laboratory parameters, the leukocyte number was found to be lower in the DF than the OFI group (P<0.001). Additionally, a higher hematocrit (P<0.001) and a lower platelet count (P<0.01) were found in the DSS group compared to the DHF or DF groups and in the DF group compared to the OFI group. Overall, 119 (61.3%) of 194 children with dengue had secondary infection. The rate of secondary infection was 40 (85.1%) of 47 cases in DSS, significantly higher than the 45 (68.2%) of 66 cases in DHF or the 34 (66.7%) of 51 cases in DF (P<0.05). In 142 confirmed DV serotypes, DV-1 and DV-2 accounted for the majority of the dengue population (43% and 39.4%, respectively), followed by DV-3 (14.1%) and DV-4 (2.1%). The prevalence of DV-2 was 19 (54.3%) of 35 cases in DSS and 14 (29.2%) of 48 cases in DF patients with statistical significance (P = 0.02).

**Table 1:** Clinical characteristics, laboratory parameters, and plasma DNA levels.

Characteristics	Controlsn = 43	OFIn = 44	DFn = 61	DHFn = 76	DSSn = 57	P†
Age (years)	8.5±3.9	6.1±4.5	9.5±3	9.9±3.3	8.3±3.3	<0.01^a^^,^ d
Female	22	18	30	37	34	0.2
Sampling day	na					
3^rd^ day of illness		19	17	18	7	0.036^b^
4^th^ day of illness		25	44	58	50	-
DV serotypes	na	na				
DV-1			21	27	13	0.5
DV-2			14	23	19	0.02^b^
DV-3			10	7	3	0.1
DV-4			3	0	0	0.09
DV-1,-4			0	1	1	-
Unknown			13	18	21	-
Secondary/Primary	na	na				
Primary			17	21	7	-
Secondary			34	45	40	<0.05^a^^,^ b
Unknown			10	10	10	-
HCT (%)*	na	38 (31–45)	41.1 (33.1–47.3)	41.2 (30–50.8)	45.1 (33.7–59.5)	<0.001^a^^,^ b ^,^ d
PLT (×10^3^/µl)*	na	150 (93–315)	114 (63–223)	94 (25.1–232)	62 (15.1–166)	<0.01^a^^,^ b ^,^ c ^,^ d
WBC (×10^3^/µl)*	na	4.4 (2.3–12)	3.3 (1.4–7.63)	3.3 (0.95–16.1)	3.6 (1.52–15.2)	<0.001^d^
DNA (ng/ml)*	143.8 (0–494.2)	158.4 (0–590.6)	322.8 (0–5890.5)	663.3 (0–14798.2)	2119.1 (49–42240.2)	<0.02^a^^,^ b ^,^ c ^,^ d ^,^ e

DF, dengue fever; DHF, dengue hemorrhagic fever; DSS, dengue shock syndrome; SD, standard deviation; na, not applicable; HCT, hematocrit, PLT, platelet, WBC, white blood cell; DV, dengue virus.

*on time of enrolment. Nineteen OFI persons' WBC were not recorded.

† Mean ± SD, Student's t test for continuous variables normally distributed; median (minimum, maximum), Mann-Whitney test for continuous variables not normally distributed; χ^2^ analysis used for categorical variables; Fisher's exact test for small expected number;

a between DHF and DSS patients;

b between DF and DSS patients,

c between DF and DHF patients,

d between DF and OFI patients,

e between control and DF.

### Circulating DNA levels measured by the PicoGreen fluorometric degradation assay

**Figure 1:**
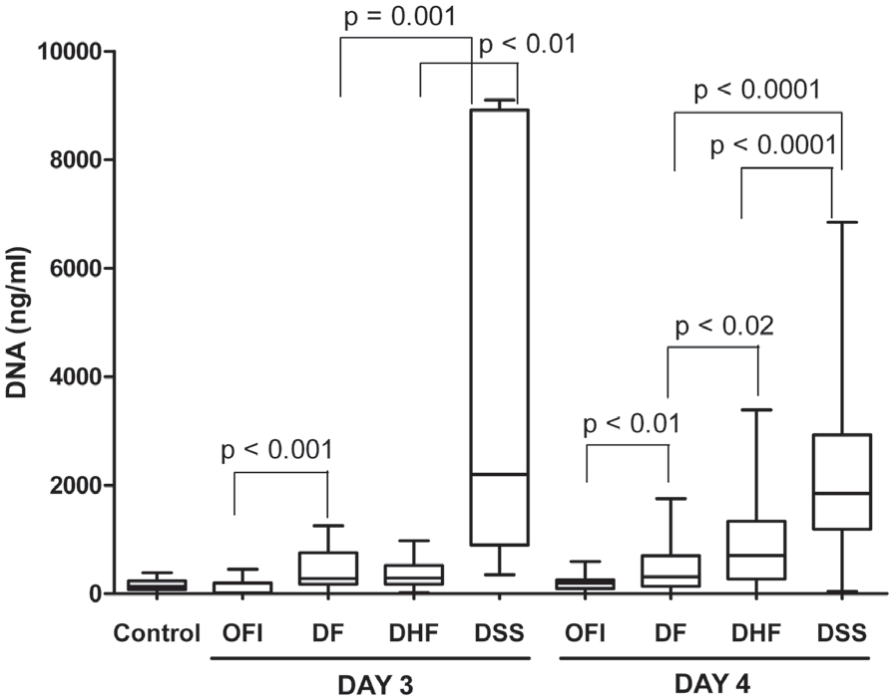
Circulating DNA levels among study groups according to the day of the blood test. Day 3 and 4 indicate the duration after the onset of high grade fever. Box-plots graphs extend from the 25^th^ to the 75^th^ percentile, and the line at the middle is the median. The error bars extend down to the lowest value and up to the highest. (The outliers are not shown). The Mann-Whitney test was used for comparisons of DNA levels between groups.

The median circulating DNA levels in the five study groups are shown in Table 1, and the distribution of the DNA values for each group according to the blood test day is shown in Figure 1. Our results showed that the median DNA levels in all dengue groups were significantly higher than those of the control and OFI groups (P<0.001). Moreover, elevated DNA levels corresponded to the severity of dengue disease: 2119.1 ng/ml in DSS, 663.3 ng/ml in DHF, and 322.8 ng/ml in DF, which were significantly different. Remarkably, circulating DNA levels were significantly higher in DSS than in DHF or DF and higher in DF than OFI, regardless of whether the blood test day was done on day 3 or 4 from the onset (Figure 1). Among the DSS population, 9 (15.8%) of 57 patients were admitted to the hospital in early stage (1–2 days before shock) whereas others entered to shock situation at the admission day. However, the DNA level was elevated early, even in 1–2 days before appearance of shock in 9 DSS cases and significantly higher in this DSS group than non-shock group (P = 0.005) (Figure S3). Additionally, 84 random samples were used for DNA extraction, followed by multiplex real-time PCR (detailed in supporting information) for nuclear DNA (nDNA) and mitochondrial DNA (mtDNA) quantification. Similarly, the median levels of nDNA and mtDNA in dengue groups were significantly higher than in the control. Increased nDNA levels were also found to be highest in DSS cases, while the mtDNA levels were not significantly different among the dengue groups (Figure S4).

### Correlation of circulating DNA levels with dengue severity, demographic, clinical, and laboratory features

Since several risk factors are reportedly associated with dengue severity, we further analyzed the correlation of circulating DNA levels with severity and these risk factors. As shown in Table 2, elevated levels of DNA positively correlated with the severity of dengue (P<0.0001), secondary infection (P = 0.0003), virulent strains of dengue virus (P = 0.04), and hematocrit (P<0.0001), but inversely correlated with platelet count (P<0.0001) and male gender (P = 0.04). In contrast, no significant correlation was observed between DNA levels and age or white blood cell (WBC) count (P>0.05).

**Table 2:** Correlation of DNA level with the severity of dengue disease and other pathological features.

Characteristics	Age	F/M	Severity	HCT	PLT	WBC	P/S	Serotype*
Correlation coefficient	−0.011	−0.12	0.63	0.35	−0.57	0.13	0.28	0.17
P value	0.85	0.04	<0.0001	<0.0001	<0.0001	0.065	0.0003	0.04
N	281	281	237	208	208	208	164	140

F/M: female/male. P/S: primary/secondary.

*serotype with increasingly virulent strain (DV-4/3/1/2). Correlation findings by Spearman rank test. Significant P values are underlined.

### Multivariate logistic regression analysis for DSS predictors

Since several variables also correlated with dengue severity, we investigated whether circulating DNA levels could independently predict DSS from non-DSS cases (DHF/DF). The results in Table 3 show that DNA levels independently correlated with DSS in the presence of age, gender, hematocrit, WBC, and platelet counts in a multivariate model (P = 0.02).

**Table 3:** Multivariate logistic regression model to predict DSS versus non-shock cases (DHF/DF).

Predictor	OR (95%CI)	P value	Adjusted OR (95%CI)	P value
Age	0.88 (0.8–0.97)	0.009	0.82 (0.7–0.94)	0.005
Gender	0.65 (0.35–1.21)	0.2	0.93 (0.4–2.1)	0.8
DNA (ng/ml)*	1.03 (1.02–1.05)	0.0	1.02 (1.003–1.04)	0.02
WBC (×10^3^/µl)*	1.08 (0.94–1.23)	0.3	0.83 (0.66–1.05)	0.1
HCT (%)*	1.21 (1.12–1.31)	0.0	1.19 (1.07–1.32)	0.001
PLT (×10^3^/µl)*	0.96 (0.94–0.97)	0.0	0.97 (0.95–0.98)	0.0

*Odds ratio represents the incremental odds of DSS for every unit increase of 100 nanogram per milliliter in DNA level, or 1000 cells per microliter in WBC, or 1 percent in HCT, or 1000 platelet per microliter in PLT.

## Discussion

In our study, cell-free circulating DNA levels were shown to be increased among dengue patients compared with control and OFI groups. This increase was highest in the DSS group, regardless of whether it was the third or fourth day of illness and even in 1–2 days before shock occurred in some DSS cases. Additionally, there was a positive correlation between DNA levels and dengue severity, as well as pathological features such as hematocrit, secondary infection, and DV serotype; but platelet count was inversely correlated. Moreover, multivariable analysis showed an independent correlation between shock syndrome and the circulating DNA level. This result suggests that the circulating DNA level could be evaluated for prognostic value in further studies. Similarly, the prognostic value of circulating DNA has been shown in patients with trauma [34] and acute stroke [35] with high sensitivity and specificity. However, the limitation of the present study is that we did not monitor the dynamic kinetics of cell-free DNA in acute phase of dengue infection and the majority of blood samples for measurement of DNA level in DSS patients were collected around the time that shock occurred. One of the common difficulties in investigating predictor for dengue severity is that the sampling time should be performed early whereas the majority of dengue patients present to hospital clinicians late during their illness, perhaps on day 3 or 4 of fever [1]. Hence, further prospective studies should be performed at primary care center for validating the benefit of circulating DNA tests in early prognosis of dengue shock syndrome.

Among various techniques established in the investigation of cell-free DNA in plasma/serum, real-time PCR is currently considered as the standard method [16], [32], although it is limited by DNA loss via binding columns during extraction. Recently, a direct fluorimetric DNA quantitative assay using the SYBR Gold dye without a purification step has been developed [36]. This assay is simple and fast, but may be affected by differential levels of proteins and particular substances. In contrast, our degradation fluorometric PicoGreen method generates a DNA-specific fluorescent signal within 90 min and uses only two small aliquots of a particular sample. Furthermore, there was a good correlation between the PicoGreen method and the standard real-time PCR (Figure S2), suggesting that PicoGreen coupled with DNase can be employed as a simple and efficient clinical method for measuring circulating DNA levels. The level of plasma DNA varies within a broad range, but the median is no more than 60 ng/ml in healthy donors according to different methods using samples after DNA purification [37]. In our direct method without extraction, the normal level of plasma DNA estimated in our study (range: 0–494.2 ng/ml) was fairly similar to that obtained using a SYBR Gold direct fluorometric assay (471±203 ng/ml). To investigate the type of DNA released into circulation during the acute phase of dengue infection, we also quantified nDNA and mtDNA in 84 samples. Both nDNA and mtDNA levels were elevated in dengue infection. The level of nDNA was associated with dengue severity, agreeing well with the circulating DNA levels measured by the PicoGreen method. In contrast, no significant difference was found between dengue groups regarding of mtDNA levels. This is probably due to the fact that the smaller mtDNA molecule (16,597 base-pairs) could be more quickly removed from circulation due to the increased vascular permeability in severe dengue cases (DHF/DSS), via the delivery systems of body, or excretion into urine compared with the larger nDNA genome (3 billion base-pairs). In addition, based on our results shown in Figure S3, the majority of circulating DNA originated from nDNA but not mtDNA.

A limiting factor of many studies of circulating DNA is that the half-life of cell-free DNA in circulation is short (4–30 min) [38], [39] due to the effects of serum nucleases, rapid removal from circulation by the liver and kidneys, and redistribution through organs and tissues [37]. Although the mechanism by which cell-free DNA is elevated in dengue patients is unknown, it is probably released from apoptotic cells, which have been found in the liver, brain, intestinal, and lung tissues during dengue infection [40]. Similarly, a study on Thailand patients found a higher rate of apoptotic PBMCs in DHF than in DF cases [41]. This DV-induced apoptotic process is triggered by both extrinsic and intrinsic pathways [42], and is thought to be involved in regulation of innate and adaptive immune responses to DV infection [25]. Moreover, failure of plasma DNA removal by the liver and kidneys due to multiple organ dysfunctions in severe dengue infections may contribute to the elevation of circulating DNA.

In our study, certain pathological features (secondary infection, hemoconcentration, and platelet count) were associated with dengue shock. These findings support a study from Pham *et al.*, which proposed that hematocrit of more than 50%, a platelet count of less than 75,000/mm^3^, and hepatomegaly were predictive features of DSS [43]. Tantracheewathorn *et al.* also found that a hemoconcentration of more than 22% and secondary infection are risk factors for DSS [44]. Another factor associated with DSS in this study is the DV-2 serotype, which is proposed to have higher virulence in comparison to other serotypes [45]. Similar results were obtained by Nguyen *et al.*, who showed that DV-2 is significantly more prevalent in DSS than DHF (70% and 45.5%, respectively) [15]. On the other hand, strong leukopenia, one of the typical manifestations of dengue disease that differentiates it from other febrile illnesses [46], was also noted among dengue patients in this study.

In conclusion, our study is the first to report an increase of cell-free DNA levels in plasma of dengue patients. Circulating DNA levels were independently associated with dengue severity. However, further large prospective studies are required to confirm the accuracy of circulating DNA levels in early prognosis of DSS.

## Supporting Information

Figure S1 **Standard curve of the fluorometric degradation method using three microliters of normal plasma with calf Lambda DNA.** The equation of the straight line is y = 2.381x, and the R^2^ is 0.999.
(TIF)

Figure S2 **Correlation between real-time PCR and the PicoGreen fluorometric degradation method.** The Spearman correlation showed an r value of 0.78, P<0.0001, n = 84.
(TIF)

Figure S3 **Levels of circulating DNA in DSS patients admitted to hospital in early stage (before shock) and in critical stage (around time of shock).** Box-plots graphs extend from the 25^th^ to the 75^th^ percentile and the line at the middle is the median. The error bars extend down to the lowest value and up to the highest. (The outliers are not shown). The Mann-Whitney test was used for comparisons of DNA levels between groups.
(TIF)

Figure S4 Levels of nuclear DNA (A) and mitochondrial DNA (B) in healthy children and patients with varying dengue severity determined by real-time PCR. Box-plots graphs extend from the 25^th^ to the 75^th^ percentile and the line at the middle is the median. The error bars extend down to the lowest value and up to the highest. (The outliers are not shown). The Mann-Whitney test was used for comparisons of DNA levels between group.
(TIF)

Table S1 **Precision and recovery of the fluorometric degradation method.**
(DOC)

Methods S1 **Detection of nuclear DNA (nDNA) and mitochondrial DNA (mtDNA) by real-time quantitative PCR.**
(DOC)
