# Supplementary figures and images for: Elevated Levels of Cell-Free Circulating DNA in Patients with Acute Dengue Virus Infection
Source: PLoS One. 2011 Oct 7;6(10):e25969. doi: 10.1371/journal.pone.0025969 (PMC3189230; doi:10.1371/journal.pone.0025969)

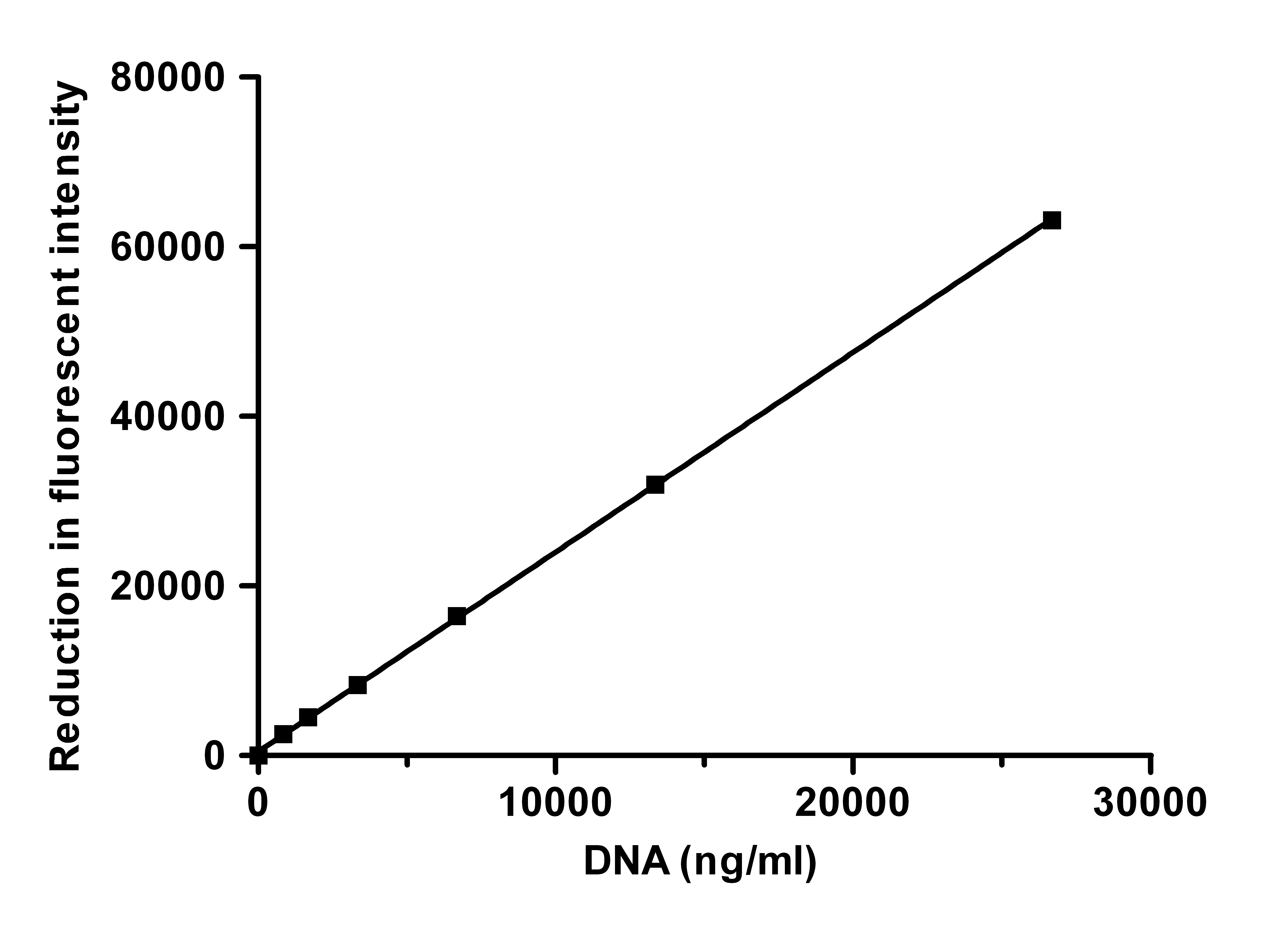

Supplement: Figure S1 — Standard curve of the fluorometric degradation method using three microliters of normal plasma with calf Lambda DNA. The equation of the straight line is y = 2.381x, and the R2 is 0.999. (TIF) [file pone.0025969.s001.tif]

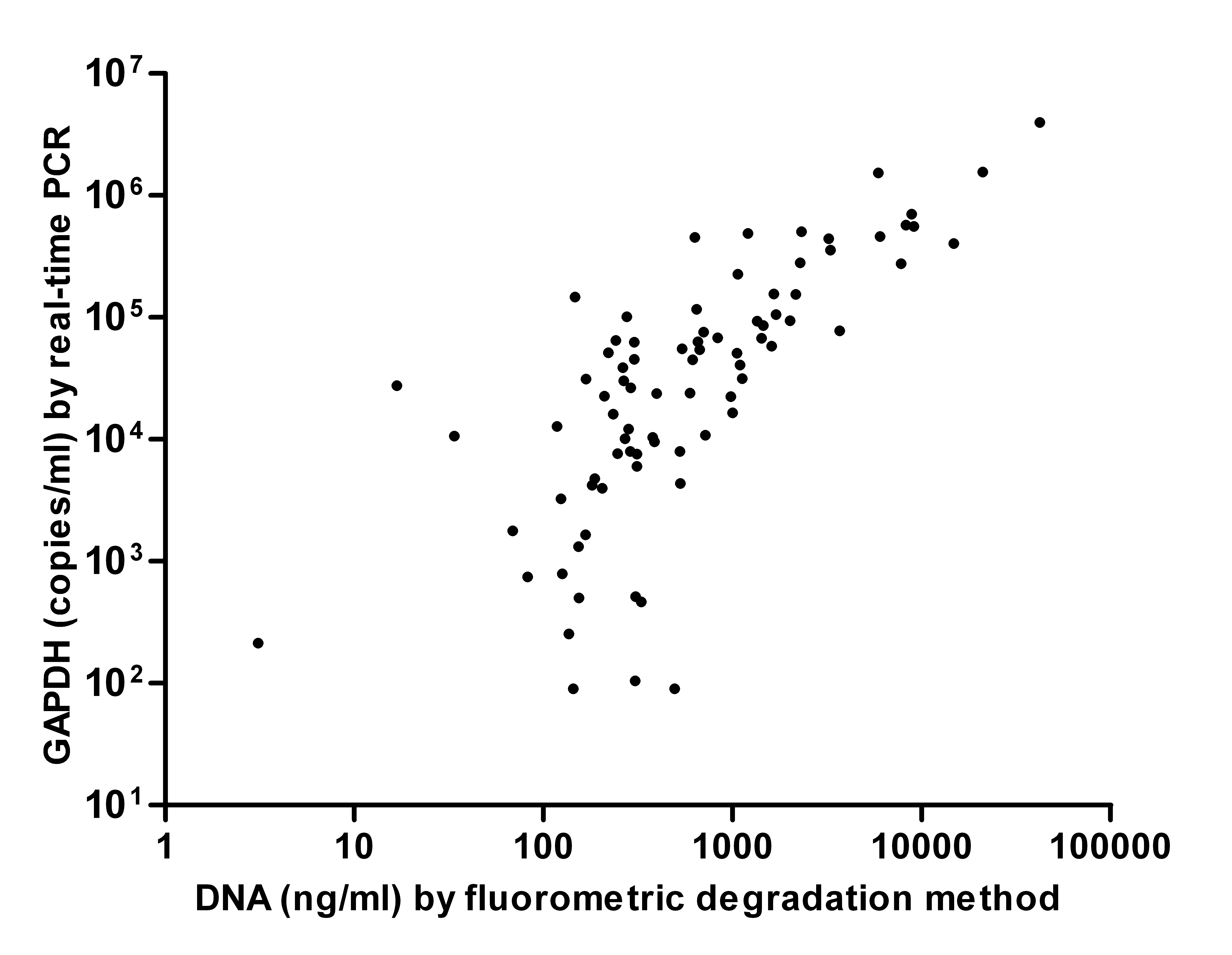

Supplement: Figure S2 — Correlation between real-time PCR and the PicoGreen fluorometric degradation method. The Spearman correlation showed an r value of 0.78, P<0.0001, n = 84. (TIF) [file pone.0025969.s002.tif]

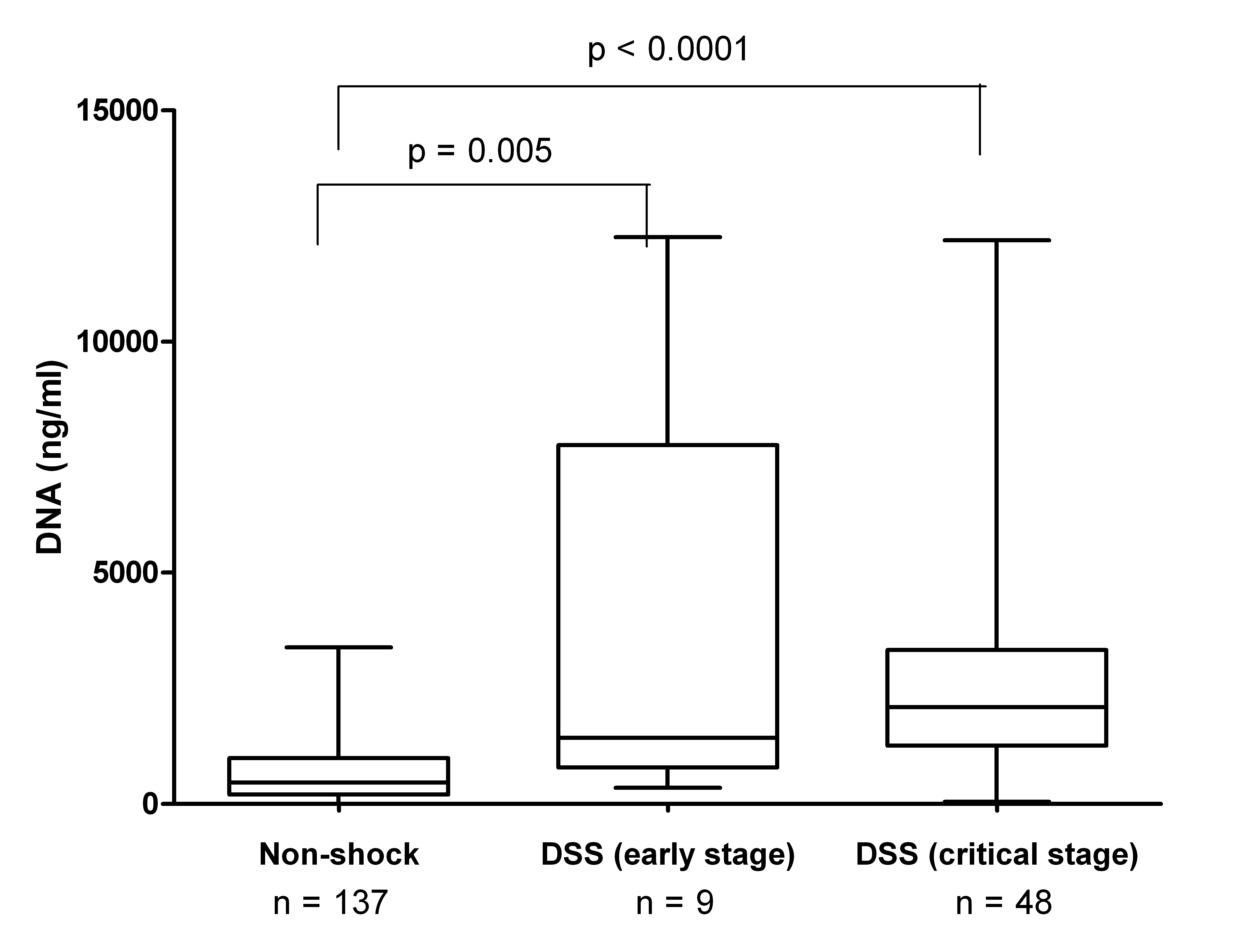

Supplement: Figure S3 — Levels of circulating DNA in DSS patients admitted to hospital in early stage (before shock) and in critical stage (around time of shock). Box-plots graphs extend from the 25th to the 75th percentile and the line at the middle is the median. The error bars extend down to the lowest value and up to the highest. (The outliers are not shown). The Mann-Whitney test was used for comparisons of DNA levels between groups. (TIF) [file pone.0025969.s003.tif]

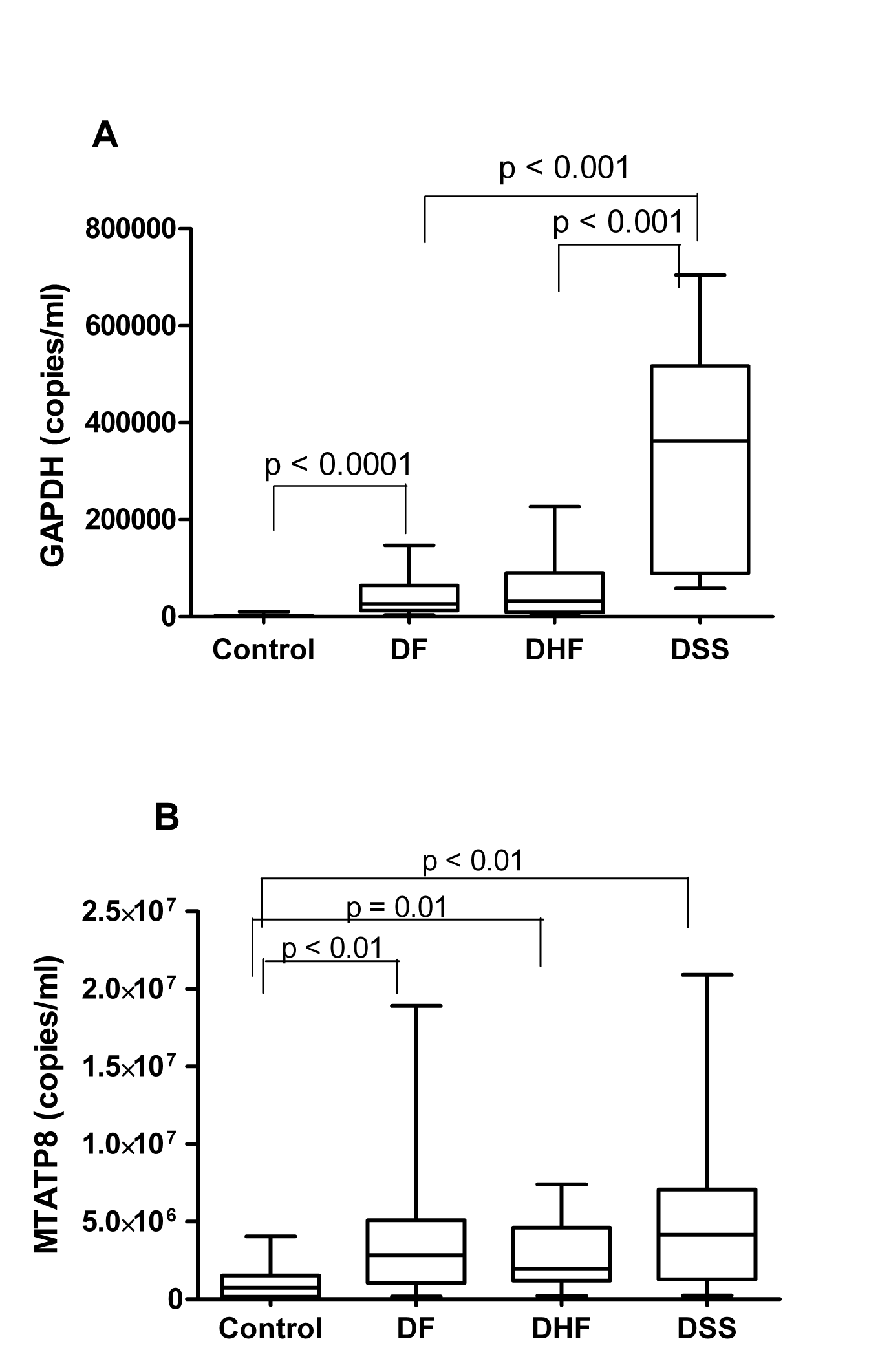

Supplement: Figure S4 — Levels of nuclear DNA (A) and mitochondrial DNA (B) in healthy children and patients with varying dengue severity determined by real-time PCR. Box-plots graphs extend from the 25th to the 75th percentile and the line at the middle is the median. The error bars extend down to the lowest value and up to the highest. (The outliers are not shown). The Mann-Whitney test was used for comparisons of DNA levels between group. (TIF) [file pone.0025969.s004.tif]
