# Supplementary material for: Elevated Levels of Cell-Free Circulating DNA in Patients with Acute Dengue Virus Infection
Source: PLoS One. 2011 Oct 7;6(10):e25969. doi: 10.1371/journal.pone.0025969 (PMC3189230; doi:10.1371/journal.pone.0025969)
Supplement: Table S1 — Precision and recovery of the fluorometric degradation method. (DOC) [file pone.0025969.s005.doc]

**Table S1.** Precision and recovery of the fluorometric degradation method

| DNA^a^  ng/ml | Within-run | | | | | | Between- run^b^ | | | | | Recovery (%) | |
| --- | --- | --- | --- | --- | --- | --- | --- | --- | --- | --- | --- | --- | --- |
|  | Mean | SD | | | CV% | | Mean | SD | | | CV% |  |  |
| 355 | 406 | | 62.9 | 15.5 | | 305 | | | 72.6 | 23.8 | | | 85.9 |
| 971 | 1114 | | 56.8 | 5.1 | | 1086 | | | 120.5 | 11.1 | | | 111.8 |
| 2200 | 2086 | | 154.4 | 7.4 | | 2029 | | | 170.4 | 8.4 | | | 92.2 |
| 4710 | 4143 | | 169.9 | 4.1 | | 4200 | | | 331.8 | 7.9 | | | 89.1 |
| 10657 | 10086 | | 332.8 | 3.3 | | 9943 | | | 258.5 | 2.6 | | | 93.3 |
| 22743 | 23114 | | 485.4 | 2.1 | | 23200 | | | 185.6 | 0.8 | | | 102.0 |

^a^ Calf Lambda DNA was added to normal plasma with a negligible DNA concentration.^b^ Assay was performed with four replicates for each concentration on four sequential days, two runs per day. SD: standard deviation, CV: coefficient of variation
