# Supplementary material for: Elevated Levels of Cell-Free Circulating DNA in Patients with Acute Dengue Virus Infection
Source: PLoS One. 2011 Oct 7;6(10):e25969. doi: 10.1371/journal.pone.0025969 (PMC3189230; doi:10.1371/journal.pone.0025969)
Supplement: Methods S1 — Detection of nuclear DNA (nDNA) and mitochondrial DNA (mtDNA) by real-time quantitative PCR. (DOC) [file pone.0025969.s006.doc]

**Supplemental Methods**

**Detection of nuclear DNA (nDNA) and mitochondrial DNA (mtDNA) by real-time quantitative PCR**

DNA was extracted from 400 μL of plasma using a commercial kit (QIAmp DNA Mini Kit; Qiagen), and was eluted in a final volume of 60 uL. The DNA was stored at -20ºC until use. Briefly, for nDNA, the glyceraldehyde-3-phosphodehydrogenase (GAPDH) housekeeping gene was used with primers 5’-CCCCACACACATGCACTTACC-3’ (forward), 5’-CCTAGTCCCAGGGCTTTGATT-3’ (reverse), and probe 5’-VIC-TAGGAAGGACAGGCAAC-MGB-3’. For mtDNA, a sequence of the mtDNA encoded ATPase (MTATP8) gene starting at locus 8446 was amplified with forward primer 5’AATATTAAACACAAACTACCACCTACC-3’, reverse primer 5’-TGGTTCTCAGGGTTTGTTATAA-3’, and 5’-6-FAM-CCTCAC

CAAAGCCCATA-MGB-3’ as the probe. Real-time PCR was performed using an ABI PRISM 7500 Sequence Detection System (Applied Biosystems, ABI) in a total reaction volume of 50 μL containing 5 μL DNA, 25 μL TaqMan® Universal PCR Master Mix, 4 primers, and 2 probes (0.6 μM for each primer and 0.2 μM for each probe), using a 2 min incubation at 50°C, an initial denaturation step at 95 °C for 10 min, and 40 cycles of 1 min at 60°C and 15 s at 95°C. Each sample was analyzed in duplicate. Two standard curves were constructed: 1)The first standard curve was generated by a dilution series of plasmids containing GAPDH or MTATP8 amplicons with a dilution factor of 10 (10 to 10^6^ copies). 2) The second standard curve was constructed by five serial dilutions of human genomic DNA with a dilution factor of 5. The copy numbers of GAPDH and MTATP8 in human samples were referred from the first standard curve (11,750 copies/μl and 503,280 copies/μl, respectively). Both standard curves showed high reproducibility with an amplification efficiency close to 1. The second standard curves for GAPDH and MTATP8 were chosen for quantification of unknown samples in this study since both are similar in nature, assuring the same behavior in the amplification reactions. The nDNA and mtDNA levels were expressed in copies per milliliter of plasma based on the following equation:

L = Q × V_DNA_ × V_PCR_^-1^ ×V_ext_^-1^ × 1000

Where L is level of nDNA or mtDNA in plasma (copies/ml); Q is quantity (copies) of nDNA or mtDNA determined by the above standard curve; V_DNA_ is the total volume of DNA obtained after extraction (60 μl per extraction); V_PCR_ is the volume of DNA solution used for PCR (5 μl); and V_ext_ is the volume of plasma extracted (400 μl).
